# Supplementary material for: Machine Learning Identification of Obstructive Sleep Apnea Severity through the Patient Clinical Features: A Retrospective Study
Source: Life (Basel). 2023 Mar 5;13(3):702. doi: 10.3390/life13030702 (PMC10056063; doi:10.3390/life13030702)
Supplement: Supplementary file 1 [file life-13-00702-s001.zip › Supplementary file S1. OSA clinical and endoscopic parameters.pdf]

**Septal Deviation Grade [20]**	2.31 ± 0.79	2.36 ± 0.76	2.26 ± 0.81	*0.160*
**Nasal Pyramid Grade [21]**	0.35 ± 0.69	0.30 ± 0.61	0.39 ± 0.75	*0.149*
**Internal Valve Collapse [22]**	0.87 ± 1.01	0.88 ± 1	0.16 ± 0.46	*<0.001*
**External Valve Collapse [23]**	0.18 ± 0.51	0.16 ± 0.46	0.20 ± 0.55	*0.387*
**Lower Turbinates Hypertrophy [24]**	2.32 ± 0.73	2.28 ± 0.73	2.37 ± 0.75	0.179
**Adenoid Hypertrophy [25]**	0.18 ± 0.51	0.2 ± 0.51	0.17 ± 0.51	*0.514*
**Friedman Tonsils Score [26]**	1.99 ± 1.17	1.92 ± 1.13	2.05 ± 1.19	*0.216*
**Mallampati Score [27]**	3.18 ± 0.71	3.15 ± 0.72	3.21 ± 0.70	*0.348*
**Friedman Palate Score [28]**	2.32 ± 0.84	2.18 ± 0.86	2.43 ± 0.80	*<0.001*
**Palate Phenotype [29]**	1.46 ± 0.69	1.43 ± 0.7	1.49 ± 0.68	*0.334*
**Endoscopic Lingual Tonsils Score [30]**	1.61 ± 0.8	1.54 ± 0.71	1.68 ± 0.79	*0.040*
**Retropalatal Mueller Maneuver [31]**	2.29 ± 1.38	2.1 ± 1.37	2.44 ± 1.36	*0.006*
**Retrolingual Mueller Maneuver [31]**	2.33 ± 0.88	2.25 ± 0.85	2.39 ± 0.89	*0.076*
**Panting Test**				
yes	25/498 (5.02%)	12/498 (2.4%)	13/498 (2.61%)	*0.692*
no	473/498 (94.97%)	208/498 (41.76%)	265/498 (53.21%)	
**Retrognathia**				
yes	99/498 (19.87%)	43/498 (8.63%)	56/498 (11.24%)	*0.868*
no	399/498 (80.12%)	177/498 (35.54%)	222/498 (44.57%)	
**Upper Jaw Contraction**				
yes	9/498 (1.8%)	3/498 (0.6%)	6/498 (1.2%)	*0.508*
no	489/498 (98.19%)	217/498 (43.57%)	272/498 (54.61%)	

Supplementary file S1. OSA clinical and endoscopic parameters.
