# Supplementary material for: Machine Learning Identification of Obstructive Sleep Apnea Severity through the Patient Clinical Features: A Retrospective Study
Source: Life (Basel). 2023 Mar 5;13(3):702. doi: 10.3390/life13030702 (PMC10056063; doi:10.3390/life13030702)
Supplement: Supplementary file 1 [file life-13-00702-s001.zip › Supplementary file S2. Mood and neurological Osa disorders .pdf]

**Morning headache**				
yes	146/498 (29.31%)	54/498 (10.84%)	92/498 (18.47%)	*0.037*
no	352/498 (70.68%)	166/498 (33.33%)	186/498 (37.34%)	
**Concentration reducted**				
yes	195/498 (39.15%)	73/498 (14.65%)	122/498 (24.49%)	*0.015*
no	303/498 (60.84%)	147/498 (29.51%)	156/498 (31.32%)	
**Anxiety/depression**				
yes	169/498 (%)	76/498 (%)	93/498 (%)	*0.947*
no	318/498 (%)	144/498 (%)	174/498 (%)	
**Irritability**				
yes	169/498 (33.93%)	163/498 (32.73%)	6/498 (1.2%)	*<0.001*
no	318/498 (63.85%)	157/498 (31.52%)	161/498 (32.32%)	
**Decreased libido**				
yes	180/498 (36.14%)	73/498 (14.65%)	107/498 (34.13%)	*0.353*
no	328/498 (65.86%)	147/498 (29.51%)	181/498 (36.34%)	

Supplementary file S2. Mood and neurological Osa disorders
