# Supplementary material for: Machine Learning Identification of Obstructive Sleep Apnea Severity through the Patient Clinical Features: A Retrospective Study
Source: Life (Basel). 2023 Mar 5;13(3):702. doi: 10.3390/life13030702 (PMC10056063; doi:10.3390/life13030702)
Supplement: Supplementary file 1 [file life-13-00702-s001.zip › Supplementary file S3. Osa comorbities.pdf]

| | | | | |
|---|---|---|---|---|
| **Familiarity for OSAS** | | | | |
| yes | 360/498 (72.28%) | 161/498 (32.32%) | 199/498 (39.95%) | *0.692* |
| no | 138/498 (27.71%) | 59/498 (11.84%) | 79/498 (15.86%) | |
| **Ipertension** | | | | |
| yes | 280/498 (56.22%) | 116/498 (23.29%) | 164/498 (32.93%) | *<0.001* |
| no | 218/498 (43.78%) | 27/498 (5.42%) | 245/498 (49.16%) | |
| **Cardiovascular disorders** | | | | |
| yes | 62/498 (12.44%) | 29/498 (5.82%) | 33/498 (6.62%) | *0.524* |
| no | 426/498 (87.56%) | 181/498 (36.34%) | 245/498 (49.16%) | |
| **Diabetes** | | | | |
| yes | 46/498 (9.24%) | 8/498 (1.60%) | 38/498 (7.63%) | *<0.001* |
| no | 452/498 (90.76%) | 204/498 (40.96%) | 248/498 (49.79%) | |
| **Dyslipidemia** | | | | |
| yes | 127/498 (25.50%) | 43/498 (8.63%) | 84/498 (16.86%) | *0.015* |
| no | 361/498 (72.48%) | 167/498 (33.53%) | 194/498 (38.95%) | |
| **COPD** | | | | |
| yes | 93/498 (18.67%) | 32/498 (6.42%) | 61/498 (12.24%) | *0.035* |
| no | 405/498 (81.32%) | 188/498 (37.75%) | 217/498 (43.57%) | |
| **Septo-Turbinoplasty** | | | | |
| yes | 112/498 (22.48%) | 48/498 (9.63%) | 64/498 (2.85%) | *0.508* |
| no | 386/498 (77.51%) | 152/498 (30.52%) | 234/498 (46.98%) | |
| **Tonsillectomy** | | | | |
| yes | 43/498 (8.63%) | 16/498 (3.21%) | 27/498 (5.42%) | *0.679* |
| no | 455/498 (91.36%) | 184/498 (36.94%) | 271/498 (54.41%) | |
| **Snoring** | | | | |
| yes | 443/498 (88.95%) | 194/498 (38.95%) | 249/498 (50%) | *<0.001* |
| no | 55/498 (11.04%) | 6/498 (1.2%) | 49/498 (9.83%) | |
| **Chocking** | | | | |
| yes | 183/498 (36.74%) | 66/498 (36.74%) | 117/498 (23.49%) | *0.005* |
| no | 315/498 (63.25%) | 154/498 (30.92%) | 161/498(32.32%) | |

**Supplementary file S3.** Osa comorbities
